# Which Neighborhood Destinations Matter in the Asian Context? The Role of Destinations in Older Adults' Physical Activity and Sedentary Behaviors

**DOI:** 10.1155/2020/8432934

**Published:** 2020-07-30

**Authors:** Jong-Hwan Park, Jung-Hoon Park, Seunghwan Song, Ting-Fu Lai, Yung Liao

**Affiliations:** ^1^Health Convergence Medicine Laboratory, Biomedical Research Institute, Pusan National University Hospital, 179, Gudeok-Ro, Seo-Gu, Busan 49241, Republic of Korea; ^2^Department of Healthcare Management, Youngsan University, 288, Junam-Ro, Yangsan, Gyeongnam 50510, Republic of Korea; ^3^Department of Thoracic and Cardiovascular Surgery, Pusan National University School of Medicine, Busan 49241, Republic of Korea; ^4^Department of Health Promotion and Health Education, National Taiwan Normal University, 162, Heping East Road Section 1, Taipei 106, Taiwan; ^5^Faculty of Sport Sciences, Waseda University, 2-579-15 Mikajima, Tokorozawa, Saitama 359-1192, Japan

## Abstract

**Background:**

Neighborhood destinations play important roles in daily activity levels of older adults. However, little is known about how specific destinations are associated with these activities and/or sedentary behaviors, especially in Asia. This study investigated how neighborhood destinations were associated with physical activity recommendations and excessive sedentary time among older adults.

**Methods:**

A telephone-based survey was conducted to collect cross-sectional data on the sociodemographic variables, residential neighborhoods, physical activities, and sedentary behaviors of 1,040 adults aged 65 years and above. Using data derived from Geographic Information Systems (GIS), an adjusted logistic regression was performed to examine the relationships between five neighborhood destination types (i.e., recreational facilities, utilitarian destinations, transit stops, temples, and schools) and both overall physical activity level and sedentary behavior.

**Results:**

Significant interactions related to physical activity and sedentary behavior were observed based on both gender and neighborhood destinations. After adjusting for potential confounders, older men living in neighborhoods containing higher numbers of temples were more likely to achieve physical activity recommendations (OR = 1.85; 95% CI: 1.16–2.96). On the other hand, older women living in neighborhoods containing higher numbers of utilitarian destinations were more likely to engage in excessive sedentary time (OR = 1.70; 95% CI: 1.12–2.56).

**Conclusions:**

In Asia, the presence of favorable local neighborhood temples may support physical activity levels for older men, while utilitarian destinations (which have previously been found to support activeness) may be related to excessive sedentary behaviors in older women.

## 1. Background

The older adult population is now increasing on a global scale. A United Nations report indicates that Taiwan is expected to be among the top 10 most-aged countries by 2050 (with more than 40% of the population aged 60 or over) [1]. A key aspect of promoting healthy and active aging in these societies is to ensure that older adults maintain the functional abilities necessary to facilitate health and well-being [2]. Research has shown that physical inactivity and sedentary behavior are the two modifiable behavioral risk factors related to daily physical function in older adults [3, 4]. In addition to the adverse impacts on physical independence, physical inactivity and prolonged sedentary time are related to higher risks of mortality and noncommunicable diseases in older adults [5, 6]. In order to make long-term changes in larger populations, it is critical to develop effective strategies for older adults through urban design and environmental initiatives.

Ecological models provide theoretical bases for understanding the role of environmental walkable attributes on physical activity [7] and sedentary behavior [8]. These models can thus inform initiatives designed to promote healthy and active ageing. In particular, neighborhoods with good access to destinations within walkable distances may support daily resident activity [9]. Previous studies have emphasized that physical activity among older adults can positively be influenced by the presence of neighborhood destinations, including utilitarian facilities (i.e., local stores and services), recreational areas (i.e., parks and sports facilities), and transit stops [9–11]. However, few studies have examined how a variety of neighborhood destination options relates to sedentary behaviors in older adults. In addition to utilitarian, recreational, and public transit destinations, neighborhood schools can also serve as public open spaces to provide opportunities for older adults to engage in daily physical activity. Based on local cultural and environmental contexts, temples can be considered distinct neighborhood destinations in a number of Asian countries, as well. Most of the Taiwanese population (around 70%) practices Buddhism, Dao, or a folk religion (i.e., superstitious beliefs) [12]. There is also a considerable density of temples in Taiwan (i.e., more than 12,000 total; one for every 2,000 people in 2018) [13, 14]. Neighborhood temples thus play a central role in everyday religious and social activity for Taiwanese seniors. We therefore hypothesized that neighborhoods containing a variety of destinations (e.g., recreational facilities, utilitarian destinations, transit stops, temples, and schools) were positively associated with physical activity levels in older adults (i.e., more physical activity resulting from trips between the home and destination), but negatively related to sedentary behavior (i.e., less sedentary time spent in the home). This study strengthened the evidence base and examined age-friendly environments in Asian contexts to investigate how a broad range of neighborhood destinations were related to physical activity levels and sedentary behaviors in older adults.

## 2. Methods

### 2.1. Participants and Procedures

A cross-sectional telephone survey using a computer-assisted telephone interviewing (CATI) technique was conducted among older Taiwanese adults in 2017. We acquired a representative sample that closely matched the characteristics of the older adult population in Taiwan using a two-phase sampling procedure. The first phase involved dividing Taiwan into four geographic areas (i.e., northern, southern, western, and eastern). The second phase involved randomly selecting respondents of the desired sex and age attributes. Well-trained interviewers (at least 8 hours of training for research ethics, questionnaire, and interviewing skills) then conducted a standardized questionnaire during each telephone survey. A total of 3,282 older adults were reached. Of these, 1,068 completed the survey (a response rate of 32.5%). No incentives were provided. Verbal informed consent was obtained at the beginning of each phone survey. All procedures used in this study were reviewed and confirmed by the Research Ethics Committee of National Taiwan Normal University (REC number: 201706HM020).

### 2.2. Self-Reported Physical Activity and Sedentary Behavior

#### 2.2.1. Physical Activity

The total amount of physical activity among older adults was assessed using the International Physical Activity Questionnaire-short version (IPAQ-SV). The test-retest reliability and criterion validity of the Taiwanese IPAQ-SV was both high (*r* = 0.78) and acceptable (*r* = 0.31–0.41) [15]. The Taiwanese IPAQ-SV is available for use via telephone survey and is thus widely utilized in phone surveys among older adult populations in Taiwan [16, 17]. Three physical activity types were determined as follows: (1) vigorous-intensity physical activity, (2) moderate-intensity physical activity (excluding walking), and (3) walking. The time spent in each of these three physical activities was calculated by multiplying frequency (i.e., how many times per week) by duration (i.e., how many hours and minutes per day). The sum indicated total physical activity. According to the recommended levels of physical activity for older adults [5], we categorized physical activity into two levels (i.e., “not achieving the physical activity recommendation (less than 150 min/week)” and “achieving the physical activity recommendation (equal to or greater than 150 min/week)”.

#### 2.2.2. Sedentary Behavior

The total time spent in sedentary behavior was measured using the validated Sedentary Behavior Questionnaire for the Elderly in Taiwan [18]. Total sedentary time was calculated for a seven-day period prior to taking the survey by adding the time spent on the following activities: screen-based sedentary time, reading, chatting with others, eating, sitting for hobbies, sitting while working or volunteering, and other sedentary activities. We categorized overall sedentary time into “less than eight hours/day” and “more than eight hours/day” using the cut-off point for heightened risk for all-cause mortality in older adults [19].

### 2.3. Objective Neighborhood Destinations

This study examined five types of neighborhood destinations (i.e., recreational facilities, utilitarian destinations, temples, schools, and public transportation). The data used for these destinations were obtained from the National Land Surveying and Mapping Center and Ministry of the Interior in Taiwan [20, 21]. Neighborhood destinations were assessed using geographic information systems (GIS) software (ArcGIS Pro; ESRI, Redlands, CA). The sum of each destination was computed for each participant's geocoded residential neighborhood. Each destination was categorized into “high” and “low” categories according to median value. The following five neighborhood destination types were revealed:

(i) Recreational facilities. The total number of parks and sports facilities. The sum of recreational facilities was categorized into “high (*N* ≥ 1)” and “low (*N* = 0).”

(ii) Utilitarian destinations. The total number of shops, convenience stores, supermarkets, post offices, libraries, book stores, restaurants, banks, and pharmacies. The sum of utilitarian destinations was categorized into “high (*N* ≥ 4)” and “low (*N* < 4).”

(iii) Temples. The total numbers of temples related to Buddhist, Daoist, and folk religions (churches and chapels were not included). The sum of temples was categorized into “high (*N* ≥ 2)” and “low (*N* < 2).”

(iv) Schools. The total numbers of elementary schools, junior high schools, high schools, colleges, and universities. The sum of schools was categorized into “high (*N* ≥ 2)” and “low (*N* < 2).”

(v) Public transportation. The total number of stations and bus stops. The sum of public transportation was categorized into “high (*N* ≥ 12)” and “low (*N* < 12).”

### 2.4. Sociodemographic Variables

Participants were asked to report their age, gender, current marital status, living status, educational level, employment status, health behaviors (i.e., smoking status, alcohol consumption, and diet), height, weight (i.e., body mass index (BMI)), and self-rated health.

### 2.5. Statistical Analyses

Data were analyzed from 1,040 respondents who had no missing data. Binary logistic regression models were used to analyze the relationships between the five types of destinations and both physical activity levels and sedentary behaviors for the total sample (adjusted for potential confounders). Likelihood ratio tests were then conducted to examine the interaction terms for the outcome variables (i.e., physical activity and sedentary behavior) between objective neighborhood destinations and gender. The sample was divided according to gender when significant interactions were found. Finally, subgroup analyses were conducted based on gender. Odds ratios and 95% confidence intervals (CIs) were computed for each variable using IBM SPSS 25.0 (significance was set at *P* < 0.05).

## 3. Results

### 3.1. Participant Characteristics

Table 1 shows the basic characteristics for the total sample and according to gender. Mean respondent age (SD) was 73.04 (± 6.13) years (50.5% were men, 64.3% were aged 65–74 years, 30.6% had tertiary degrees, 10.3% had full-time jobs, 75.9% were married, 85.7% were living with others, 7.0% were current smokers, 9.7% consumed alcohol, 82.3% had healthy diets, 12.3% reported poor health status, and 52.8% were of normal-weight). A total of 79.3% respondents completed at least 150 minutes of weekly physical activity, while 30.9% engaged in daily sedentary behavior for more than eight hours. Chi-square tests revealed that older men were more likely to be married, have full-time jobs, tertiary educations, smoke, and consume alcohol.

### 3.2. Objective Neighborhood Destinations Associated with Physical Activity and Sedentary Behavior (Total Sample)

For the total sample, older adults living in neighborhoods with higher numbers of temples were more likely to engage in physical activity adding up to at least 150 minutes/week (OR = 1.71; 95% CI: 1.21–2.41). Older adults living in neighborhoods with greater numbers of utilitarian destinations were more likely to engage in sedentary time lasting more than eight hours/day (OR = 1.48; 95% CI: 1.12–1.95) (Table 2).

### 3.3. Interactions between Gender and Objective Neighborhood Environment

Significant interactions relating to physical activity were observed between gender and temples (*P* = 0.03). Significant interactions relating to sedentary behavior were found between gender and utilitarian destinations (*P* = 0.04) (Table 3).

### 3.4. Objective Neighborhood Destinations Associated with Physical Activity and Sedentary Behavior in Older Men and Women

A gender stratification revealed that neighborhoods with higher numbers of temples were positively associated with the achievement of physical activity recommendations in older men (OR = 1.85; 95% CI: 1.16–2.96). On the other hand, neighborhoods with higher numbers of utilitarian destinations were related to excessive sedentary time in older women (OR = 1.70; 95% CI: 1.12–2.56) (Tables 4 and 5).

## 4. Discussion

This is the first study to examine a range of objective neighborhood destinations and their relationships with both physical activity levels and sedentary behaviors among older populations in an Asian context. Results revealed that different neighborhood destinations had specific behavioral effects according to gender. This is consistent with previous findings [17]. Our results showed that a higher number of neighborhood temples aided older men in meeting their daily physical activity recommendations, while a greater number of utilitarian destinations was associated with excessive sedentary time in older women. These findings may provide two critical implications for urban policy and planning initiatives designed to promote “Active Aging” in Asian countries. First, neighborhood temples should be considered prominent local destinations for promoting daily physical activity levels for older men. Second, although previous studies have found that utilitarian destinations were related to increased walking time [9, 22] (in the Asian context, see [23, 24], walking-supportive environmental attributes may increase sedentary behavior for older adults in Asian countries.

This study uniquely found that higher numbers of neighborhood temples were positively associated with older men meeting the recommended 150 minutes of total weekly physical activity. It is traditionally assumed that men and women have distinct gender roles in a number of Asian cultures (e.g., Japanese, Korean, and Chinese). Here, women are more likely to be responsible for housework [25, 26]. On the other hand, older men may have more free time. Here, temples may provide a “Third Place (social surroundings separate from the usual social environments)” [27] in which older men can engage in social events and religious activities in the Taiwanese cultural context. Easy access to neighborhood temples can thus motivate older men to engage in increased physical activity while traveling to these locations from home. Previous studies have found that environmental settings (e.g., shopping malls [28] and parks [29]) can be used for community-level physical activity programs or interventions. Our results also suggest that neighborhood temples can serve as important environmental settings for effective community-based physical activity interventions among older men. In this regard, urban planners in Taiwan may consider how religious spaces can be used to support aging populations.

Contrary to our hypothesis, we also found that neighborhoods with more utilitarian destinations were associated with excessive sedentary time among older women. This is consistent with previous findings in the Asian context asserting that walkable neighborhood attributes were positively associated with sedentary behaviors [23, 24]. Here, it is possible that neighborhoods with higher numbers of utilitarian destinations reduce the time it takes older women to complete daily errands. Such individuals would thus have increased time to engage in sedentary behaviors. First, these results suggest that the possible negative impacts of favorable neighborhood destinations on sedentary behavior should be considered when planning intervention programs. Second, an increasing number of studies are finding different environmental/behavioral associations between Western and Asian countries. Our results thus suggest the importance of further examining these relationships in the Asian context.

This study had several limitations. First, it employed a cross-sectional design that may have limited the causal inferences between neighborhood destinations and active/sedentary behaviors among older adults. Second, respondents self-reported their physical activities and sedentary behaviors. Responses were thus subject to recall bias. Further studies should thus attempt to objectively measure these factors among older adults. Finally, older adults in Taiwan may be reluctant to report their exact residential addresses [23]. The neighborhood destinations used in this study were thus obtained according to participant residential neighborhood rather than exact residential addresses. Nevertheless, residential neighborhood units have widely been used as validated geographic areas when measuring walkability attributes [30].

## 5. Conclusions

Gender is a potential moderator between neighborhood destinations and physical activity/sedentary behavior among older adults. In Asia, conveniently located neighborhood temples may support older men in reaching their daily physical activity requirements, while utilitarian destinations (which have previously been found as activity-supportive attributes) may be related to excessive sedentary behavior among older women.

## Figures and Tables

**Table 1:** Characteristics of the study participants (*N* = 1,040).

	Total sample (*N* = 1040)		Older men (*N* = 525)		Older women (*N* = 515)		
	*N*	%	*N*	%	*N*	%	*P* value^a^
Age group (years)							0.26
65-74	669	64.3%	329	62.7%	340	66.0%	
75+	371	35.7%	196	37.3%	175	34.0%	
Marital status							<0.001^∗^
Married	789	75.9%	428	81.5%	361	70.1%	
Unmarried	251	24.1%	97	18.5%	154	29.9%	
Employment status							0.001^∗^
Full-time job	107	10.3%	71	13.5%	36	7.0%	
No full-time job	933	89.7%	454	86.5%	479	93.0%	
Educational level (years)							<0.001^∗^
<13	722	69.4%	336	64.0%	386	75.0%	
≥13	318	30.6%	189	36.0%	129	25.0%	
Living status							0.83
Alone	149	14.3%	74	14.1%	75	14.6%	
With others	891	85.7%	451	85.9%	440	85.4%	
Current smoking status							<0.001^∗^
Yes	73	7.0%	66	12.6%	7	1.4%	
No	967	93.0%	459	87.4%	508	98.6%	
Alcohol consumption							<0.001^∗^
Yes	101	9.7%	89	17.0%	12	2.3%	
No	939	90.3%	436	83.0%	503	97.7%	
Healthy diet							0.04^∗^
Yes	856	82.3%	420	80.0%	436	84.7%	
No	184	17.7%	105	20.0%	79	15.3%	
BMI (kg/m^2^)							0.52
Normal weight	549	52.8%	272	51.8%	277	53.8%	
Not normal weight	491	47.2%	253	48.2%	238	46.2%	
Self-rated health							0.07
Good	493	47.4%	266	50.7%	227	44.1%	
Fair	419	40.3%	194	37.0%	225	43.7%	
Poor	128	12.3%	65	12.4%	63	12.2%	
Physical activity							0.49
150+ min/week	825	79.3%	412	78.5%	413	80.2%	
<150 min/week	215	20.7%	113	21.5%	102	19.8%	
Sedentary behavior							0.35
8+ hours/day	321	30.9%	169	32.2%	152	29.5%	
<8 hours/day	719	69.1%	356	67.8%	363	70.5%	

^a^Chi-square tests.

^∗^ *P* < .05.

**Table 2:** Associations of objectively measured neighborhood destinations with physical activity and sedentary behavior in the total sample.

	Odds of meeting physical activity recommendation			Odds of excessive sedentary time		
	OR	95% CI	*P*	OR	95% CI	*P*
Recreational facilities						
Low	1.00 (ref.)			1.00 (ref.)		
High	1.06	0.77-1.47	0.72	1.32	0.98-1.77	0.07
Utilitarian destinations						
Low	1.00 (ref.)			1.00 (ref.)		
High	1.01	0.74-1.39	0.92	1.48	1.12-1.95	0.006^∗^
Temple						
Low	1.00 (ref.)			1.00 (ref.)		
High	1.71	1.21-2.41	0.002^∗^	1.10	0.76-1.34	0.96
Schools						
Low	1.00 (ref.)			1.00 (ref.)		
High	1.23	0.90-1.68	0.19	1.17	0.89-1.53	0.27
Public transportation						
Low	1.00 (ref.)			1.00 (ref.)		
High	0.91	0.66-1.24	0.54	1.21	0.92-1.59	0.18

Adjusted for gender, age, current marital status, living status, educational level, employment status, smoking status, alcohol consumption, healthy diet, BMI, and self-rated health. ^∗^Statistically significant (*P* < .05).

**Table 3:** Statistical significance of the interactions between gender and variables related to destinations using binary logistic regression models.

Objective neighborhood destinations	*P* value for interaction term with gender	
	Physical activity	Sedentary behavior
	*P* value	*P* value
Recreational facilities	0.82	0.15
Utilitarian destinations	0.81	0.04^∗^
Temple	0.03^∗^	0.92
Schools	0.35	0.38
Public transportation	0.72	0.20

Adjusted for age, current marital status, living status, educational level, employment status, smoking status, alcohol consumption, healthy diet, BMI, and self-rated health.

^∗^Statistically significant (*P* < .05).

**Table 4:** Associations of the objectively measured neighborhood destinations with physical activity by gender.

	Odds of meeting physical activity recommendation					
	Older men			Older women		
	OR	95% CI	*P*	OR	95% CI	*P*
Temple						
Low	1.00 (ref.)			1.00 (ref.)		
High	1.85	1.16-2.96	0.01^∗^	1.52	0.91-2.54	0.11

Adjusted for age, current marital status, living status, educational level, employment status, smoking status, alcohol consumption, healthy diet, BMI, and self-rated health.

^∗^Statistically significant (*P* < .05).

**Table 5:** Associations of the objectively measured neighborhood destinations with sedentary behavior by gender.

	Odds of excessive sedentary behavior				
	Older men			Older women		
	OR	95% CI	*P*	OR	95% CI	*P*
Utilitarian destinations						
Low	1.00 (ref.)			1.00 (ref.)		
High	1.33	0.91-1.97	0.14	1.70	1.12-2.56	0.01^∗^

Adjusted for age, current marital status, living status, educational level, employment status, smoking status, alcohol consumption, healthy diet, BMI, and self-rated health.

^∗^Statistically significant (*P* < .05).

## Data Availability

The dataset supporting the conclusions of this article is available in the laboratory of Dr. Yung Liao (corresponding author).
